# TCM Database@Taiwan: The World's Largest Traditional Chinese Medicine Database for Drug Screening *In Silico*

**DOI:** 10.1371/journal.pone.0015939

**Published:** 2011-01-06

**Authors:** Calvin Yu-Chian Chen

**Affiliations:** 1 School of Chinese Medicine, China Medical University, Taichung, Taiwan; 2 Department of Bioinformatics, Asia University, Taichung, Taiwan; 3 Department of Computational and Systems Biology, Massachusetts Institute of Technology, Cambridge, Massachusetts, United States of America; Griffith University, Australia

## Abstract

Rapid advancing computational technologies have greatly speeded up the development of computer-aided drug design (CADD). Recently, pharmaceutical companies have increasingly shifted their attentions toward traditional Chinese medicine (TCM) for novel lead compounds. Despite the growing number of studies on TCM, there is no free 3D small molecular structure database of TCM available for virtual screening or molecular simulation. To address this shortcoming, we have constructed TCM Database@Taiwan (http://tcm.cmu.edu.tw/) based on information collected from Chinese medical texts and scientific publications. TCM Database@Taiwan is currently the world's largest non-commercial TCM database. This web-based database contains more than 20,000 pure compounds isolated from 453 TCM ingredients. Both cdx (2D) and Tripos mol2 (3D) formats of each pure compound in the database are available for download and virtual screening. The TCM database includes both simple and advanced web-based query options that can specify search clauses, such as molecular properties, substructures, TCM ingredients, and TCM classification, based on intended drug actions. The TCM database can be easily accessed by all researchers conducting CADD. Over the last eight years, numerous volunteers have devoted their time to analyze TCM ingredients from Chinese medical texts as well as to construct structure files for each isolated compound. We believe that TCM Database@Taiwan will be a milestone on the path towards modernizing traditional Chinese medicine.

## Introduction

For thousands of years, traditional Chinese medicine (TCM) holds an important role in medical diagnosis and treatments in Eastern Asia. However, due to lack of systematic investigation and poor understanding of TCM regimens, TCM is less recognized in the Western society. Recently, increasing effort has been devoted to study TCM, from which a large number of bioactive compounds have been isolated and studied. Hence, there is a need to establish a database to organize the enormous amounts of TCM data and make the virtual screening of TCM ingredients easily accessible.

Although there are many websites detailing information for TCM sources, traditional usage from ancient material medica texts, and processing and storage procedures, these databases, such as The Chinese medicine sampler (http://www.chinesemedicinesampler.com) and Dictionary of Chinese Herbs (http://alternativehealing.org), contain little information of TCM ingredients at molecular level. The TCMGeneDIT database [1] is an effective search engine for TCM-related literature, but the information on the TCM constituents is not well organized. Other databases, including TCMD [2], Chinese Traditional Medicinal Herbs Database [3], and TCM-ID [4], provide general TCM information and 3D structures of TCM ingredients. However, these databases are either inaccessible or highly restricted for information sharing. To date, the ZINC database [5] is currently the largest free 3D molecule database. However, there is no TCM related database similar to the scale of ZINC yet. In the hope of building a complete TCM ingredient library, we construct the TCM Database@Taiwan (http://tcm.cmu.edu.tw/).

In the past, our laboratory has performed screening for multiple TCM components and has successfully discovered novel lead compounds, such as anti-viral, anti-inflammation, anti-cancer, stroke prevention compounds, and hypnotic medications [6]–[17]. By constructing TCM Database@Taiwan, we can further facilitate the virtual screening process in the experiment design for the TCM lead drug discovery. We firmly believe that the constituents from TCM are the sources to derive novel pharmaceutical compounds.

## Results and Discussion

TCM Database@Taiwan (http://tcm.cmu.edu.tw) is currently the largest non-commercial TCM database available for download. This web database is designed in both English and traditional Chinese languages. All 3D structures are constructed in mol2 format and are readily used for virtual screening.

### TCM organization

Right now, the database contains 20,000 ingredients from 453 different herbs, animal products, and minerals TCM regimens. In the near future, the database will further record folk herb ingredients. The TCM database is organized by proposed actions of Chinese medicines (Table S1). There are a total of twenty-two different drug classes and some are further divided into subclasses based on clinical applications recorded in TCM monographs. It should be noted that our TCM classification is based on traditional Chinese theories including the Yin-yang, the human Meridan/Channel system, the Five Elements theory, and the Zang Fu organ theory. It should also be noted that TCM listed under certain classes, such as parasites elimination, dampness reduction and itchiness relief medicinal, and topical application medicinal, contain toxic ingredients and are no longer prescribed in clinics. These ingredients are present in the TCM database for the completeness of TCM records. These data do not imply endorsement for any clinical or private use of the toxic TCM compounds nor for any animal products present in the database.

### Search and Display

**Figure 1:**
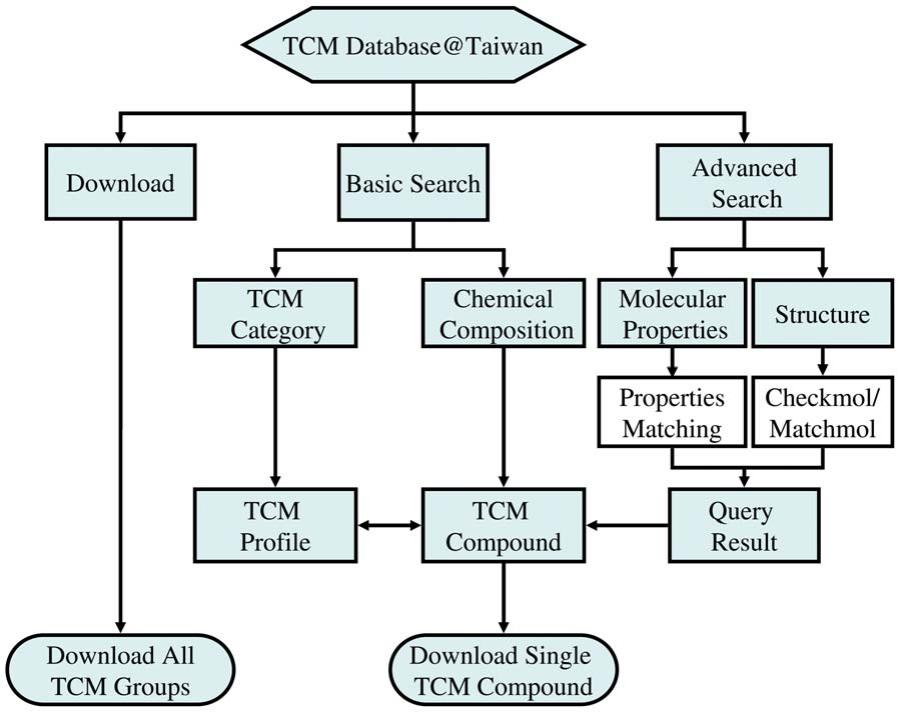
Search flowchart and available download options in TCM database.

**Figure 2:**
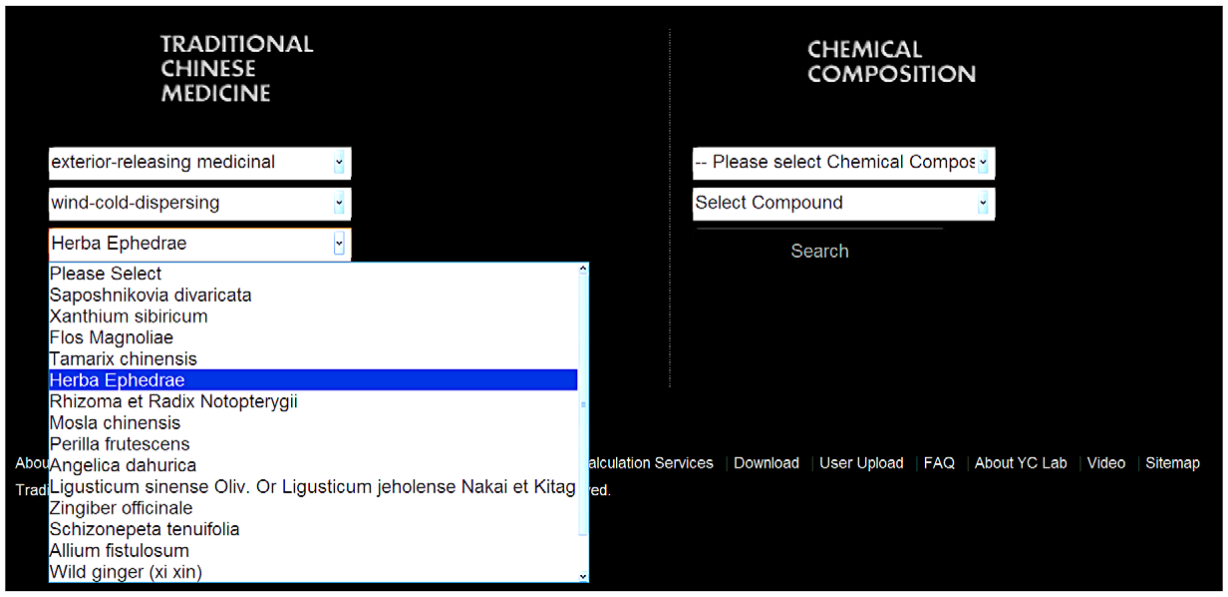
The search tool available in the TCM database. (a) search by TCM category and (b) search by chemical composition.

An overview of available search options and download options is shown in Figure 1. TCM Database@Taiwan can be browsed by simple and advanced search options. The simple search option enables users to browse the website either by TCM medicine or by TCM ingredients (Figure 2). The search-by-TCM-medicine option allows users to select the intended drug action group and then the desired TCM medicine. The search-by-TCM-chemical-composition option allows users to find possible TCM sources of the given molecule. This search result is directly linked to the TCM compound webpage without the need to specifying TCM action group.

For each search options, the result is organized in a “TCM profile” that provides the identified TCM compound(s) and the associated references. Clicking on a TCM compound in the profile will display the compound's 2D and 3D structures, as well as its molecular properties in a new page. Users can download the structure of the molecule in cdx (2D) or mol2 (3D) format at the bottom of the TCM compound webpage. In addition, users can also click on the links to browse other Chinese medicines that contains the selected TCM compound.

**Figure 3:**
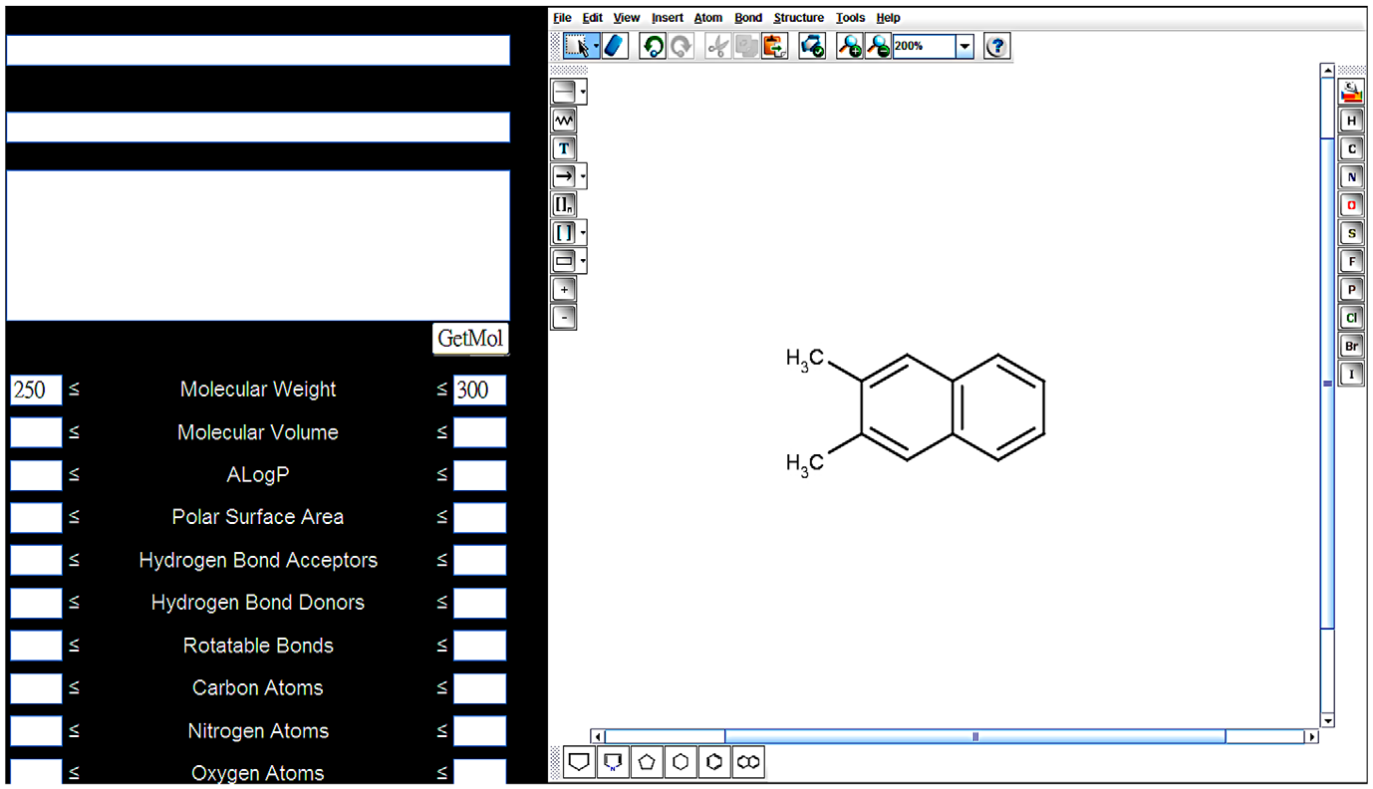
The advance search interface.

The advanced search option incorporates a molecular drawing interface (ChemAxon) for structure search (Figure 3). Users can also specify structure types, including exact search and substructure search, whichever best describes users' needs. Moreover, users can perform searches by specifying the molecular properties, such as molecular weight and ALogP. Both of the advanced search options can be used alone or in conjunction. The result will return molecule(s) that satisfy the input specification in a tabular format with name and 2D representation. For users who prefer search by Chinese medicine names and other TCM information, a website search engine is available at top of every page.

### Download Interface

The TCM ingredients are organized by their drug action classes and their original sources. Users can download a specific drug class or a specific TCM or the whole TCM database on Download page.

### Sharing TCM Constituents

As technology advances, new TCM ingredients have been isolated and studied each day. Therefore, we implemented an upload function for scientists who are interested in sharing their findings on Chinese medicines. Users may upload their own molecules to the TCM database server in mol2 format. The uploaded molecules will be reviewed and incorporated into the TCM database.

Overall, TCM Database@Taiwan is constructed in the hope to create the most complete TCM library and to strengthen the TCM research network to date. In addition, this web-based database is implemented with virtual screening and molecular simulation functions. For both biochemists studying TCM and medicinal chemists designing novel lead compounds, this database serves as a useful resource for virtual screening as well as for the references in biochemical assays. In the future, we will incorporate genetic algorithm (GA) and support vector machine (SVM) for further TCM classifications based on molecular properties. We expect the construction of TCM Database@Taiwan to become a milestone for modern TCM researches.

## Methods

The TCM herbs, animal products and minerals listed in the TCM Database @Taiwan were originated from Chinese medical texts and dictionaries [18]–[22]. In addition, the TCM constituents were collected manually from published results available on Medline [23] and ISI Web of Knowledge (http://apps.isiknowledge.com). The data were organized into twenty-two major classes based on their proposed therapeutic actions recorded in Chinese medical texts [24]–[26]. The 2D and 3D structures of TCM constituents were built by ChemBioOffice 2008 (CambridgeSoft, Cambridge, MA). The 3D structures were energy minimized in MM2 force field. Physicochemical properties, including ALogP and polar surface area, were calculated using ChemBioOffice.

With efficiency and stability as our main concern, we implemented our database on a Linux server, then use MySQL5.0, Apache, and hypertext preprocessor (PHP) for database and web server development. For friendly user interfaces, we used ChemAxon MarvinView applet (https://www.chemaxon.com) for 2D and 3D representation. In addition, we applied MarvinSketch tool from ChemAxon for structure drawing in the advanced search option.

**Figure 4:**
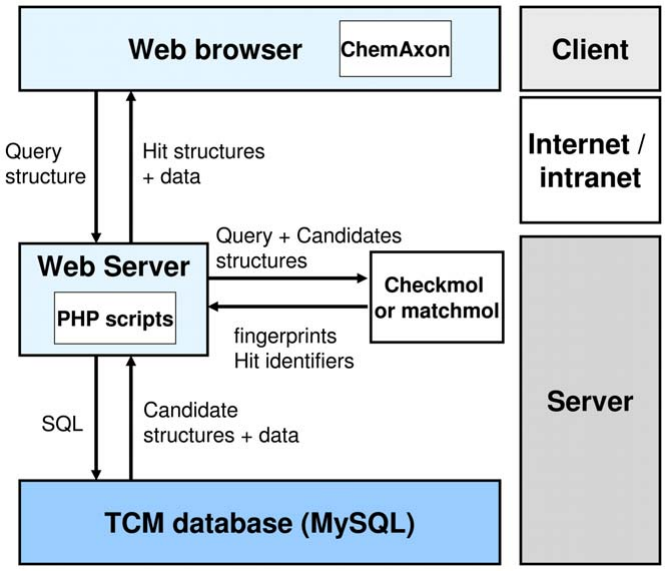
The infrastructure of the TCM database. The design is adapted from Norbert Haider's MolDB5R.

In the advanced search section, the search engine was developed based on the Norbert Haider's MolDB5R package [27]. The infrastructure of the advanced search engine is shown in Figure 4. For search-by-specifying-molecular-properties, a search script written in PHP is used to search and retrieve TCM compounds that satisfied all the input properties. Two programs, checkmol and matchmol, are used to perform the background structure searching and matching process. The queried structure is read by the checkmol program to generate molecular descriptors, which is then used in the preliminary search in the TCM database. The matchmol program is responsible for full structure comparison of the input structures from checkmol. For additional structure search options, such as substructure search, the matchmol program treat one compound as “needle” and other molecule(s) as “haystack” to determine the best-fit substructure of another structure. All the search options in the advanced search section can be used in conjunction. By specifying both molecular properties and query structure scaffold, an additional data screening process is performed to ensure that all the input criteria are met.

## Supporting Information

Table S1 Summary of medicines present in each TCM class. The English translation of each class is taken from WHO publication.
(DOC)
