# Supplementary material for: TCM Database@Taiwan: The World's Largest Traditional Chinese Medicine Database for Drug Screening In Silico
Source: PLoS One. 2011 Jan 6;6(1):e15939. doi: 10.1371/journal.pone.0015939 (PMC3017089; doi:10.1371/journal.pone.0015939)
Supplement: Table S1 — Summary of medicines present in each TCM class. The English translation of each class is taken from WHO publication. (DOC) [file pone.0015939.s001.doc]

Table S1. Summary of medicines present in each TCM class. The English translation of each class is taken from WHO publication.

| **Main classes** | **Sub-classes** | **No.^a^** |
| --- | --- | --- |
| Exterior-releasing medicinal | Wind-cold-dispersing (16) | 28 |
|  | Wind-heat dispersing (12) |  |
| Tonifying and replenishing medicinal | Qi-tonifying (15) | 62 |
|  | Yang-tonifying (23) |  |
|  | Blood-tonifying (7) |  |
|  | Yin-tonifying (17) |  |
| Astringent medicinal | Anhidrotic (3) | 17 |
|  | Lung-intestine astringent medicinal(8) |  |
|  | Securing essence, reducing urination and checking vaginal discharge (6) |  |
| Wind-dampness dispelling medicinal | Wind-dampness dispelling and cold dispersing (13) | 26 |
|  | Wind-dampness dispelling and heat clearing (8) |  |
|  | Wind-dampness dispelling and bone(sinew) strengthening (5) |  |
| Dampness-resolving medicinal |  | 9 |
| Interior-warming medicinal |  | 13 |
| Worm-expelling medicinal |  | 9 |
| Emetic medicinal |  | 3 |
| Parasites elimination, dampness reduction and itchiness relief medicinal |  | 8 |
| Topical application medicinal |  | 6 |
| Liver-pacifying and wind-extinguishing medicinal | Liver-yang calming (7) | 15 |
|  | Extinguishing wind to arrest convulsions (8) |  |
| Orifice-opening medicinal |  | 7 |
| Heat-clearing medicinal | Heat-clearing and fire-purging (13) | 64 |
|  | Heat-clearing and dampness-drying (10) |  |
|  | Heat-clearing and detoxicating (30) |  |
|  | Heat-clearing and blood-cooling (6) |  |
|  | Deficiency heat clearing medicinal (5) |  |
| Purgative medicinal | Offensive purgative medicinal (4) | 13 |
|  | Laxative medicinal (2) |  |
|  | Drastic (purgative) water-expelling (7) |  |
| Water-draining medicinal | Water-draining and swelling-dispersing(11) | 27 |
|  | Water-draining and strangury-relieving (11) |  |
|  | Water-draining and anti-icteric (5) |  |
| Qi-regulating medicinal |  | 22 |
| Digestant medicinal |  | 8 |
| Hemostatic medicinal | Blood-cooling hemostatic medicinal (9) | 36 |
|  | Stasis-resolving hemostatic medicinal (5) |  |
|  | Astringent hemostatic medicinal (9) |  |
|  | Meridian-warming hemostatic medicinal (3) |  |
| Blood-activating and stasis-resolving medicinal | Blood-activating analgesic medicinal (7) | 33 |
|  | Blood-activating menstruation regulating (11) |  |
|  | Blood-activating trauma-curing (9) |  |
|  | Blood-breaking mass-eliminating (6) |  |
| Cough-suppressing and panting-calming medicinal | Cold-phlegm resolving (8) | 34 |
|  | Heat-phlegm resolving (15) |  |
|  | Cough-suppressing and panting-calming (11) |  |
| Tranquillizing medicinal | Settling and tranquillizing (3) | 9 |
|  | Heart-nourishing tranquillizing (6) |  |
| Anti-malaria medicinal |  | 4 |

^a^The number of medicines found in each functional class.
